# Dental implant site preparation with conventional rotary drill or piezosurgery: five-year after placement results from a within person randomised controlled trial

**DOI:** 10.1186/s40729-024-00582-7

**Published:** 2024-12-18

**Authors:** Adriano Azaripour, Vittorio Siro Leone Farina, Marco Esposito, Jacopo Buti, Bilal Al-Nawas, Keyvan Sagheb

**Affiliations:** 1 Department of Oral and Maxillofacial Surgery, University Medical Center, Johannes Gutenberg University, Augustusplatz 2, 55131 Mainz, Germany; 2 Department of Periodontology and Restorative Dentistry, University Medical Center, Johannes Gutenberg University, Mainz, Germany; 3 Private Practice, Bergamo, Italy; 4 Dental School, IRCCS San Raffaele Scientific Institute and Vita-Salute San Raffaele University, Milan, Italy; 5 Unit of Periodontology, UCL Eastman Dental Institute, London, UK

**Keywords:** Dental implants, Implant site preparation, Piezoelectric surgery, Rotary drills

## Abstract

**Purpose:**

To evaluate whether there are clinical benefits by preparing dental implant sites using piezosurgery instead of conventional rotary drills in healed bone crests and if initial crestal soft tissue thickness could have an impact on marginal bone loss.

**Methods:**

Twenty-five partially edentulous patients requiring two single implants in molar/premolar areas had each site randomly allocated to either piezosurgery or to conventional rotary drill preparation according to a split-mouth design. Definitive screw-retained metal-ceramic crowns were delivered after 6 months. All patients were followed to 5 years after placement. Outcome measures were: implant/crown failures, complications, peri-implant marginal bone level changes, resonance frequency analysis (RFA), and time required to complete site preparation, recorded, when possible, by blinded assessors.

**Results:**

No patients dropped-out and no implant failed. Five years after placement, there were no statistically significant differences for complications (only one complication in the piezo group: difference = 0.04; P = 1), for peri-implant bone loss (difference = −0.11 mm; 95% CI −0.24 to 0.01; P = 0.083), and for RFA changes (6 months) (difference = −0.35; 95% CI −1.95 to 1.25; P = 0.672 between groups). Significantly more time was needed to prepare implant sites with piezosurgery (difference = 236.8 s; 95% CI −286.12 to −187.48; P < 0.0001). Initial soft tissue thickness had no effect on peri-implant bone loss (estimate = 0.05; 95% CI −0.03; 0.12; P = 0.239).

**Conclusions:**

No clinically appreciable differences were noticed when placing implants using piezosurgery or conventional instrumentation with rotary drill, however, the preparation with rotary drills was on average 4 min faster. No effect of initial crestal soft tissue thickness was observed on peri-implant bone loss.

## Introduction

Among the numerous ongoing debates in implant dentistry, there is also the one about which could be the best way to prepare implants sites for placing dental implants. Traditionally, implant sites were prepared by a sequence of drills of increased diameters, the last diameter depending on the implant diameter, the quality of bone and the wished insertion torque. Despite that rotary drills are almost universally used for implant site preparation, some authors suggested the use of piezosurgery instead, either alone [1] or in combination with rotary drills [2]. The hypothesis behind is that piezosurgery may induce a more precise cut as well as inducing less heating, therefore decreasing the risk of bone necrosis and improving bone healing compared to rotary drills [3]. At the same time, piezosurgery can be less aggressive than rotary drills if a nerve is accidentally hit. On the other hand, an animal study showed a tendency for more bone to implant contact and RFA, believed to corresponds to increased implant stability, at drilled sites when compared to implant sites prepared with piezosurgery [4]. In addition, drilling required a 4-min shorter preparation time and two implants did not integrated in the piezo group versus one in the drilled group [4]. Another ex-vivo study failed to show any statistically significant differences between drilling and piezosurgery regarding heat generation and implant stability assessed with the resonance frequency analysis (RFA) [5]. However, site preparation with piezosurgery took significantly longer time than with rotary drills. Therefore, it would be interesting to know whether a better clinical outcome could be obtained by using piezoelectric surgery instead of conventional drills to prepare dental implant sites.

The aims of this RCT were primarily to evaluate clinical outcome of preparing implant sites with piezosurgery compared to conventional drilling, and secondarily to evaluate if initial crestal soft tissue thickness could have an impact on marginal bone loss. The present article is reported according to the CONSORT statement (http://www.consort-statement.org/) and its extension checklist for reporting within person randomised trials (http://www.consort-statement.org/extensions/overview/withinperson) to improve the quality of reports of within person randomised controlled trials.

## Materials and methods

### Trial design

This was a single-centre randomised controlled trial (RCT) of split-mouth design and blind assessment. Each patient received two identical implants (one test and one control implant): test implants were placed after having prepared the site with a piezoelectric device while control implants were placed in sites prepared with conventional drills.

### Patient selection

Any patient requiring at least two single implant-supported crowns in molar or premolar areas (wisdom teeth excluded), being at least 18 years old and able to understand and sign an informed consent form was eligible for inclusion. The two implant sites could be adjacent and had to allow the placement of two implants 11 mm long and 4.0 mm wide, i.e., they had to have a bone height of at least 12 mm and a width of at least 7 mm. For patients with more than two suitable implant sites, the operator chose those two sites with the most similar characteristics at the screening visit. The operator coded the selected sites as implant site number 1 and implant site number 2.

Exclusion criteria were:

- General contraindications to implant surgery;
- Systemic diseases (i.e. such as uncontrolled diabetes, cardiovascular disease, and immunocompromised conditions were excluded to ensure a Homogeneous study population)
- Immunosuppressed or immunocompromised patients;
- Irradiation in the head and/or neck area;
- Pregnancy or lactating;
- Smokers;
- Untreated periodontitis;
- Poor oral hygiene and motivation (full-mouth plaque and bleeding scores less or equal to15%);
- Substance abusers;
- Psychiatric disorders;
- Acute infection or suppuration at any of the sites intended for implant placement;
- Need of any type of bone augmentation at implant placement;
- Post-extractive sites (implants could only be inserted after at least 6 months of healing);
- Unable to commit to 5-year follow-up;
- Under treatment or had previous treatment with intravenous amino-bisphosphonates;
- Patients referred only for implant placement if the follow-up could not be conducted at the treatment centre;
- Participation in other clinical studies if the present protocol could not be fully adhered to.

The study protocol was approved by the University of Mainz Ethics Committee (Ethics Committee No.: 837.1 85.1 5 (9953). Patients were recruited and treated by AA at the Department of Operative Dentistry and Periodontology of the University Medical Center (Mainz, Germany) using similar and standardised procedures. Prior to enrolment, all patients were asked to read, and once understood, to sign an informed consent form to document that they understood the scope of the study (including procedures, follow-up evaluations, and any potential risks involved), were allowed opportunities to ask questions pertaining to this study, and were apprised of treatment alternatives. All procedures were performed in accordance with the principles outlined in the Declaration of Helsinki. The study was open to any qualifying patients without regard to sex or race.

### Clinical procedures

**Fig. 1:**
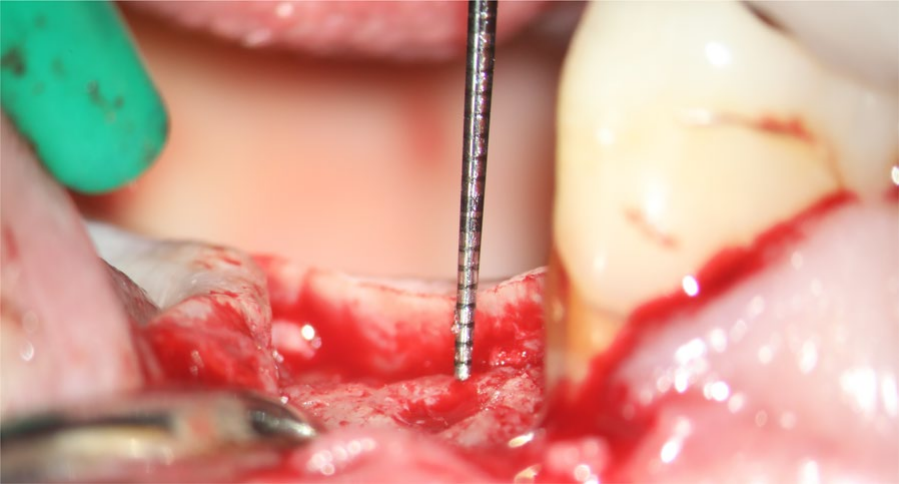
Measurement of the mucosa height

Preoperative panoramic radiographs were taken. Patients received a single dose of prophylactic antibiotic 1 h prior to the intervention: 1 g of amoxicillin or 600 mg of clindamycin, if allergic to penicillin. Patients rinsed with chlorhexidine mouthwash 0.2% for 1 min prior to the intervention. Patients were treated under local anaesthesia using articaine with adrenaline 1:100.000. After crestal incision, flap elevation was performed first vestibulary, and the thickness of the supracrestal mucosa was measured with a periodontal probe at the incision site (Fig. 1). Thereafter the lingual/palatal flap was raised. The sequentially numbered sealed envelope corresponding to the patient recruitment number was opened and implant site number 1 was treated according to the content of the envelope. Consequently, implant site number 2 was treated with the other procedure, according to a split-mouth design. The two study implants were placed in the same surgical session following similar procedures and were restored simultaneously with similar single crowns.

**Fig. 2:**
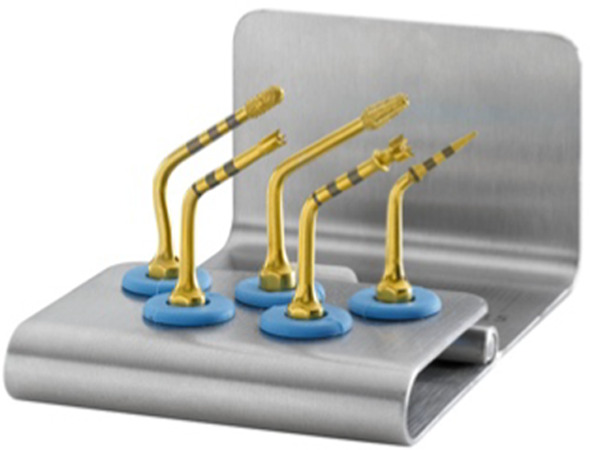
Sequence of piezoelectric inserts used to prepare the test implant sites

**Fig. 3:**
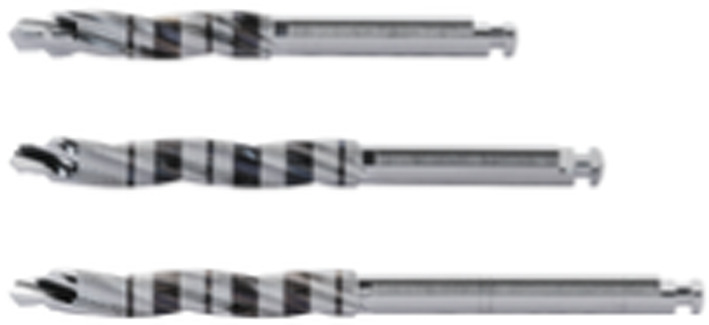
Sequence of drills used to prepare the control implant sites

**Fig. 4:**
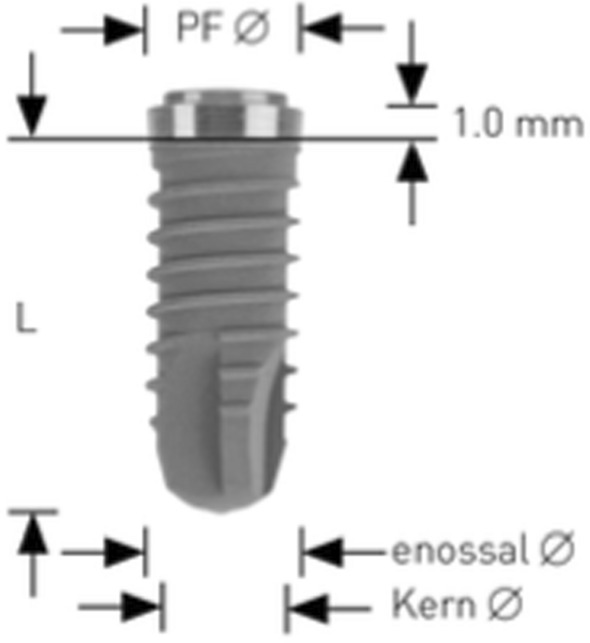
Illustration showing the implant design used in the study

**Fig. 5:**
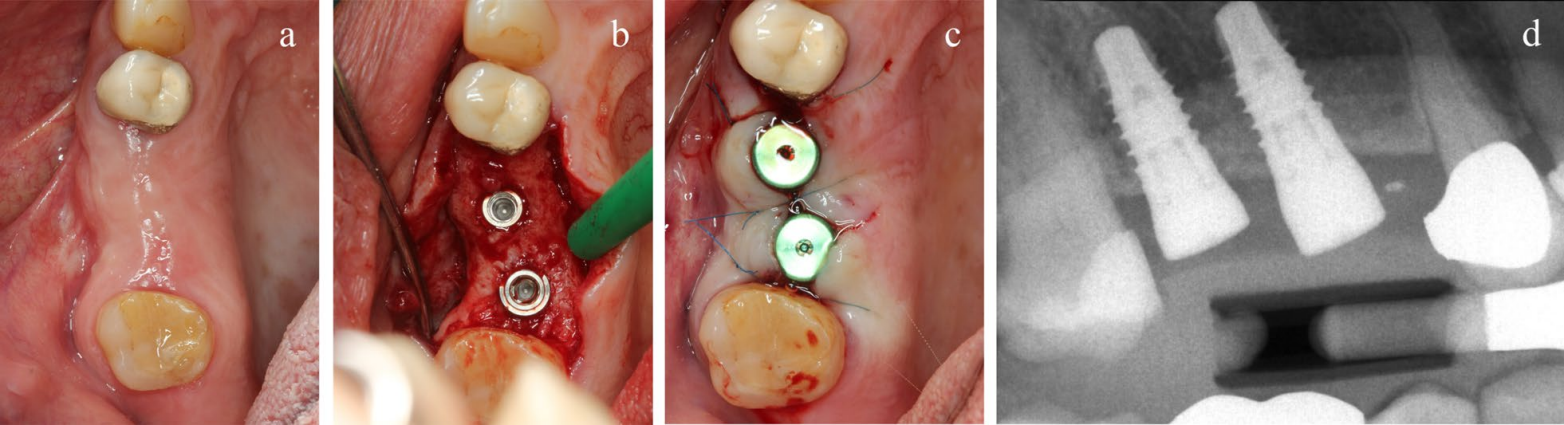
**a**-**c**: **a** preoperative view of a representative patient; **b** site 15 was randomly allocated to piezosurgery and site 16 to conventional drilling; **c** flap closure; **d** postoperative baseline periapical radiograph

Implant sites, randomly allocated to piezo instrumentation, were prepared using piezo-electric device (PIEZOSURGERY touch, Mectron, Cherasco, Italy), starting with a special tip for the initial preparation (IM1S Mectron) followed by IM2, IM3, IM3-4, P3-4 tips by Mectron; Fig. 2). Control sites per prepared using a sequence of conventional drills (Fig. 3) as described by the manufacturer (VECTODrill Thommen Medical, Grenchen, Switzerland). Cylindrical SPI Element INICELL (Thommen) titanium grade 4 implants with a polished collar of 1 mm height and internal flat to flat hexagon connection were used (Fig. 4). All implants were 11 mm long by 4 mm in diameter. Implants were placed by setting the motor with a torque of 30 Ncm. The neck of the implant was placed flush to the surrounding bone. At this point implant stability was measured by a blinded assessor (K.S) using the Osstell Mentor RFA device (Osstell, Integration Diagnostics, Goteborg, Sweden) using the dedicated transducers (SmartPeg, Osstell) for the SPI 4.0 mm connection. All implants were measured twice (from mesio-distal and bucco-lingual directions). Finally, healing abutments were connected and flaps were sutured with 6.0 sutures (Premilene B/Braun Aesculap, Tuttlingen, Germany) around the abutments. Baseline periapical radiographs were taken **(**Fig. 5a-d) and if the peri-implant marginal bone levels were difficult to be evaluated another periapical radiograph was taken. Ibuprofen 600 mg was prescribed to be taken thrice a day during meals, for 3 days. In case of stomach problems or allergy to non-steroidal anti-inflammatory drugs, 1 g of paracetamol was recommended instead. Patients were instructed to use 0.12% chlorhexidine mouthwash for one minute thrice a day for 1 week, and to avoid brushing and possible trauma on the surgical sites. After 1 week, patients were checked, sutures were removed and oral hygiene instructions were delivered.

**Fig. 6:**
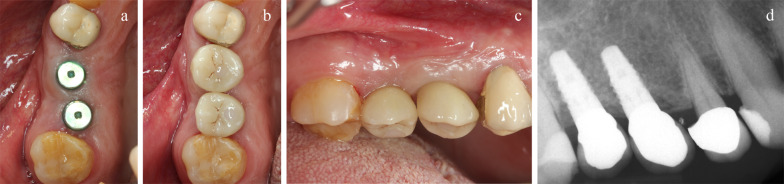
**a**-**d**: **a** clinical situation prior to delivery of definitive crowns; **b** occlusal and **c** vestibular view at delivery of definitive crowns; **d** periapical radiograph at initial loading (6 months after implant placement)

Implants were left to heal unloaded for 6 months (Fig. 6a), and six months after surgery, implant level digital impressions were taken, screw-retained metal-ceramic crowns were fabricated on customised titanium abutments and delivered within 2 weeks (Fig. 6b and c). Periapical radiographs were taken (Fig. 6d), and oral hygiene instructions were delivered. Exactly the same procedures were implemented at both implants during the same sessions. Patients were recalled for maintenance every 3 months for the entire duration of the study.

### Outcome measures

This study tested the null hypothesis that there were no differences in clinical outcome between the two procedures against the alternative hypothesis of a difference.

**Fig. 7:**
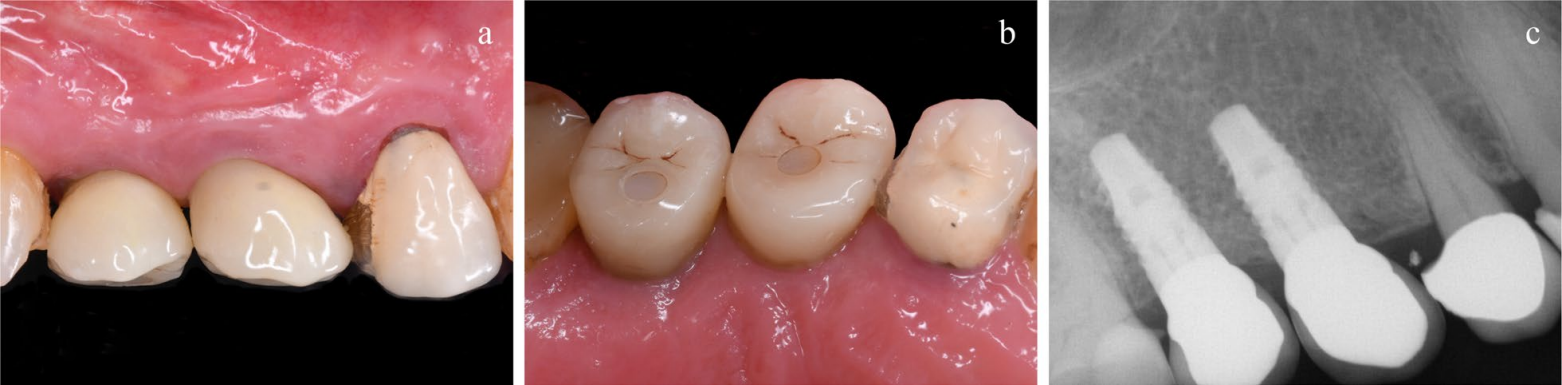
**a**-**c**: **a** and **b** clinical and **c** radiographic images at 5 years after placement

Outcome measures were:

- Implant/crown failures: implant mobility, removal of stable implants dictated by progressive marginal bone loss or infection, and any mechanical complications rendering the implant not usable (e.g. implant fracture) were considered implant failures. If a definitive crown had to be replaced for any reason, it accounted as a crown failure. The stability of individual implants was assessed clinically by attempting to rock the crown with the metal handles of two dental instruments at each follow-up visit.
- Any biological or biomechanical complications. Examples of biological complications are fistula and peri-implantitis. Examples of biomechanical complications are loosening or fracture of the abutment screw.
- Peri-implant marginal bone level changes evaluated on digital periapical radiographs taken with the paralleling technique at implant placement, 1 (Fig. 5d), 3, 6 (Fig. 6d), 12, 24 months and at 5 years (Fig. 7a-c) after initial implant placement. In case of an unreadable radiograph, a second radiograph was obtained. Peri-implant marginal bone levels were measured using the Planmeca software (Helsinki, Finland). The software was calibrated for every single image using the known implant diameter. Measurements of the mesial and distal bone crest level adjacent to each implant were made to the nearest 0.01 mm. Reference points for the linear measurements were the coronal margin of the implant collar and the most coronal point of visible bone-to-implant contact. The measurements at mesial and distal sides of each implants were averaged at implant level and then at group level.
- Resonance frequency analysis (RFA): Stability of individual implants was also measured with Osstell Mentor RFA device (Osstell, Integration Diagnostics) using the dedicated transducers (SmartPeg, Osstell) for the SPI 4.0 mm connection. All implants were measured twice (from mesio-distal and bucco-lingual directions and the two measurements were averaged) at implant placement, 1, 3 and 6 months after implant placement.
- Time needed to prepare the implant site: it was calculated in seconds by a dental assistant starting from the use of the first osteotomy instrument to the complete seating of the implant.

A blind outcome assessor (K.S.) assessed implant stability (RFA) and another blinded assessor (V.F.) measured marginal bone levels. Both were not involved in the clinical treatment. The blinding process was maintained throughout the study by ensuring that the assessors were unaware of the surgical method used for each implant site. The assessors are trained specialist in implantology who conducted all measurements independently. Complications were handled and reported directly by the responsible clinician who was not blinded.

### Sample size, randomisation and allocation concealment

A sample size was estimated in 47 implants, given an effect size d = 0.487065, α err prob 0.05, and power (1-ß err prob = 0.90). Effect size was determined based on a previous similar study reporting ISQ values of 75.7 ± 5.2 in the piezosurgery and 73.3 ± 4.6 in the conventional drilling group at 3 months [2]. Due to the split-mouth design of the study, patients provided both test (piezosurgery) and control (conventional drilling) implants. In order to avoid underpowered results (< 90%), unbalanced groups and to account for possible drop-outs, three implants were added, scoring a total sample size of 50 implants (25 patients).

One computer generated restricted randomisation lists was created. Only one investigator (K.S.), who was not involved in the selection and treatment of the patients, knew the random sequence and had access to the random list stored in a password protected portable computer. The random codes were enclosed in sequentially numbered, identical, opaque, sealed envelopes. After flap elevation, the envelope corresponding to the patient recruitment number was opened, and implant site number 1 was allocated to the group determined by the content of the envelope, and other site received the alternative intervention. Therefore, treatment allocation was concealed to the investigators in charge of enrolling and treating the patients.

### Statistical analysis

All data analysis was performed according to a pre-established analysis plan by a dentist (JB) with expertise in statistics who analysed the data without knowledge of the group codes. The implant sites were the statistical unit of the analyses. Differences between the groups in crown/implant failures and complications (dichotomous outcomes) were compared using a McNemar test. Between-group differences for continuous outcomes (mean marginal bone level and RFA) at different time points were estimated by paired t-test. Comparisons between the various follow-up endpoints and the baseline measurements were made by paired t-tests, to detect any changes in mean marginal bone level for each study group. Two-level (patient and implant) mixed effect models with patient as random effect for each time point after implant placement with baseline (implant placement) as a covariate were created to estimate between-group differences for mean marginal bone level and RFA changes from baseline. A further two-level (patient and implant) mixed effect model with patient as random effect was created to evaluate the soft tissue thickness as predictor of marginal bone level changes over time adjusted for baseline ISQ values. All statistical comparisons were conducted at the 0.05 level of significance.

## Results

**Fig. 8:**
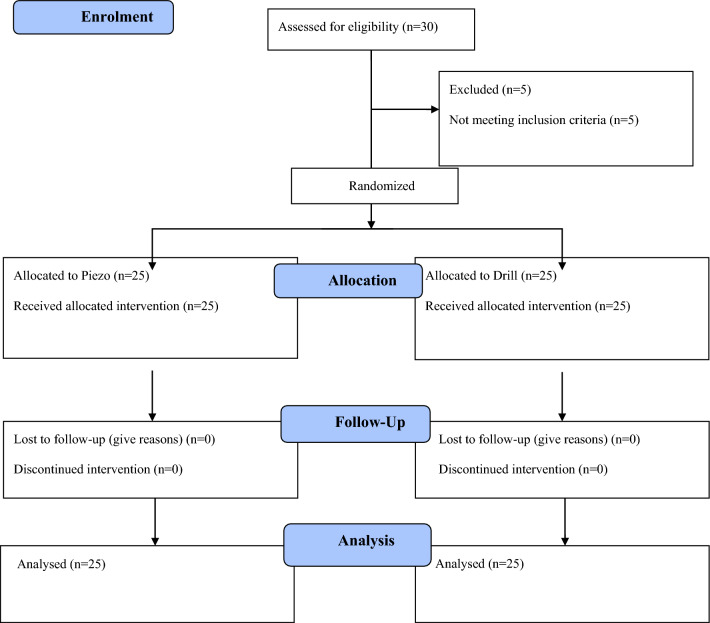
CONSORT 2010 Flow Diagram

Thirty patients were screened and 25 patients were consecutively enrolled in this trial. Five patients were not included because they did not have sufficient bone volumes to receive 11 × 4 mm implants. All patients were treated according to the allocated interventions. No patient dropped-out and data from all timepoints could be collected. The data of all patients were evaluated in the statistical analyses. No deviations from the protocol were reported (Fig. 8).

Patients were recruited and received the implants from May 2015 to May 2016. The follow-up of all patients was to 5 years after implant placement. There were 14 males and 11 females, with a mean age of 47.2 years (range 32 to 73). Implant site characteristics by study groups are described in Table 1. There were no apparent significant baseline imbalances between the two groups.

- Crowns and implant failures: No implant or crown failed.
- Complications: Only one complication occurred at one implant of the piezo group (crown screw loosening). There was no statistically significant difference in number of patients experiencing complications between the two groups (difference in proportions = 0.04; P (McNemar test) = 1).
- Peri-implant marginal bone levels (Table 2) and changes (Table 3). At implant insertion time (baseline), peri-implant marginal bone levels were 0.49 ± 0.31 mm at piezo sites and 0.41 ± 0.22 mm at drilled sites, the difference being not statistically significant (difference = −0.08 mm; 95% CI −0.21 to 0.06; P (paired t-test) = 0.2577; Table 2). Statistically significant differences in favour of drilled sites were observed at 3 months (0.17 mm), 6 months, 1, 2 and 5 years after implant placement (0.14 mm, at each time intervals; Table 2).

**Table 1:** Implant site characteristics

	Piezo N = 25	Drill N = 25
Implants in first premolar position	1 (4%)	1 (4%)
Implants in second premolar position	6 (24%)	2 (8%)
Implants in first molar position	15 (60%)	13 (52%)
Implants in second molar position	3 (12%)	9 (36%)
Implants in third molar position	0 (0%)	0 (0%)
Implants in maxillae	12 (48%)	12 (48%)
Implants in mandibles	13 (52%)	13 (52%)
Mucosa thickness at crestal level [Mean (SD)] in mm	2.9 (0.82)	2.98 (0.86)

**Table 2:** Mean radiographic peri-implant marginal bone levels and differences between groups and time periods up to 5 years after implant placement

	Implant placement	1 month	3 months	6 months	1 year	2 years	5 years
	N Mean (SD) [95% CI]	N Mean (SD) [95% CI]	N Mean (SD) [95% CI]	N Mean (SD) [95% CI]	N Mean (SD) [95% CI]	N Mean (SD) [95% CI]	N Mean (SD) [95% CI]
Piezo	25 0.49 (0.31) [0.36;0.61]	25 0.78 (0.36) [0.64;0.93]	25 0.98 (0.35) [0.84;1.13]	25 1.06 (0.37) [0.91;1.22]	25 1.25 (0.36) [1.11;1.40]	25 1.26 (0.35) [1.11;1.40]	25 1.23 (0.33) [1.09;1.37]
Drill	25 0.41 (0.22) [0.32;0.50]	25 0.65 (0.36) [0.50;0.80]	25 0.81 (0.34) [0.67;0.95]	25 0.92 (0.39) [0.76;1.08]	25 1.11 (0.38) [0.96;1.27]	25 1.12 (0.37) [0.96;1.27]	25 1.09 (0.35) [0.94;1.23]
Difference [95% CI]	−0.08 [−0.21;0.06]	−0.13 [−0.28;0.01]	−0.17 [−0.32;−0.02]	−0.14 [−0.27;−0.01]	−0.14 [−0.28;−0.01]	−0.14 [−0.28;−0.01	−0.14 [−0.28;−0.01
P-value	0.2577	0.0725	0.0266*	0.0359*	0.0455*	0.0459*	0.0459*

^*^Statistically significant difference between groups

**Table 3:** Mean radiographic peri-implant marginal bone level changes between groups and time periods up to 5 years after implant placement

	Implant placement – 1 month	Implant placement—3 months	Implant placement—6 months	Implant placement—1 year	Implant placement—2 years	Implant placement—5 years
	N Mean (SE) [95% CI]	N Mean (SE) [95% CI]	N Mean (SE) [95% CI]	N Mean (SE) [95% CI]	N Mean (SE) [95% CI]	N Mean (SE) [95% CI]
Piezo	25 0.30 (0.07) [0.15;0.44]	25 0.50 (0.08) [0.33;0.67]	25 0.58 (0.07) [0.42;0.73]	25 0.77 (0.08) [0.60;0.94]	25 0.77 (0.08) [0.60;0.94]	25 0.74 (0.08) [0.58;0.91]
Drill	25 0.24 (0.07) [0.11;0.38]	25 0.40 (0.08) [0.24;0.56]	25 0.51 (0.07) [0.35;0.66]	25 0.70 (0.07) [0.55;0.86]	25 0.71 (0.07) [0.56;0.86]	25 0.68 (0.07) [0.53;0.82]
Difference* [95% CI]	−0.09 [−0.22; 0.04]	−0.16 (−0.31;−0.01)	−0.10 [−0.21;0.01]	−0.11 [−0.23;0.01]	−0.11 [−0.23;0.01]	−0.11 [−0.24;0.01]
P-value*	0.178	0.045**	0.082	0.091	0.092	0.083

^*^Two-level mixed effect with patient as random effect at 1, 3 and 6 months, 1, 2 and 5 years after implant placement with baseline (implant placement) as a covariate

^*^*Statistically significant difference between groups. All changes from baseline (implant placement) statistically significant (P < 0.05)

At 5-year post-placement peri-implant bone loss was 0.74 ± 0.08 mm at piezo sites and 0.68 ± 0.07 mm at drilled sites, the difference being not statistically significant (difference = −0.11 mm; 95% CI −0.24 to 0.01; P (paired t-test) = 0.083; Table 3). Only at 3 months post-placement was noticed a statistically significant difference in favour of drilled sites (difference = −0.16 mm; 95% CI −0.31 to −0.01; P (paired t-test) = 0.03; Table 3). Marginal bone loss from baseline was statistically significant in both groups (P (paired t-test) <0.05; Table 3).

Soft tissue thickness did not result to be statistically significantly affecting marginal bone level changes (estimate = 0.05; 95% CI −0.03; 0.12; P = 0.239) over time in the mixed effect model adjusted for baseline ISQ values (Table 4).

- Resonance frequency analysis (RFA) values (Table 5) and changes (Table 6). At implant insertion time (baseline), RFA values were 70.98 ± 10.38 at piezo sites and 70.82 ± 9.21 at drilled sites, the difference being not statistically significant (difference = −0.16; 95% CI −3.61 to 3.29; P (paired t-test) = 0.9245; Table 5). At 6 months post-placement, RFA change was 3.94 ± 2.11 at piezo sites and 3.7 ± 2.06 at drilled sites, the difference being not statistically significant (difference = −0.35; 95% CI −1.95 to 1.25; P (paired t-test) = 0.672; Table 6). RFA changes from baseline were not statistically significant in both groups (Table 6).
- The average time needed to prepare the implant site was of 329.44 ± 134.65 s for the piezo group and 92.64 ± 52.60 s for the drill group, showing a statistically significant difference in favour of the conventional drilling procedure (difference = −236.8 s; 95% CI −286.12 to −187.48; P < 0.0001).

**Table 4:** Mixed effect model for mean radiographic peri-implant marginal bone level changes between groups and time periods up to 5 years after implant placement with soft tissue thickness as baseline covariate

Term	Estimate	SE	95% Lower	95% Upper	P-value
Intercept	0.77	0.26	0.26	1.27	0.0030*
ISQ at implant placement	0.001	0.003	−0.01	0.01	0.8454
Soft tissue thickness	0.05	0.04	−0.03	0.12	0.2393
Time point (1 month)	−0.22	0.03	−0.28	−0.2	< 0.0001*
Time point (3 months)	−0.04	0.03	−0.1	0.02	0.1710
Time point (6 months)	0.05	0.03	−0.01	0.11	0.1112
Time point (12 months)	0.24	0.03	0.18	0.31	< 0.0001*
Time point (24 months)	0.25	0.03	0.19	0.31	< 0.0001*
Time point (5 years)	0.22	0.03	0.16	0.28	< 0.0001*
Site preparation with piezo	0.07	0.01	0.04	0.10	< 0.0001*

Two-level mixed effect with patient as random effect with baseline ISQ values, soft tissue thickness as a covariates; and site preparation method and time points as factors

^*^Statistically significant estimate

**Table 5:** Mean RFA values between groups and time periods up to 6 months after implant placement

	Implant placement	1 month	3 months	6 months
	N Mean (SD) [95% CI]	N Mean (SD) [95% CI]	N Mean (SD) [95% CI]	N Mean (SD) [95% CI]
Piezo	25 70.98 (10.38) [66.69;75.27]	25 69.05 (16.26) [62.33;75.75]	25 73.96 (10.16) [69.76;78.16]	25 74.82 (9.74) [70.90;78.94]
Drill	25 70.82 (9.21) [67.02;74.62]	25 70.68 (13.48) [65.12;76.24]	25 73.4 (12.87) [68.09;78.71]	25 74.52 (12.61) [69.32;79.72]
Difference [95% CI]	25 −0.16 [−3.61;3.29]	25 1.65[−1.30;4.58]	25 −0.56 [−2.42;1.30]	25 −0.4[−2.23;1.43]
P-value	0.9245	0.2615	0.5397	0.6554

**Table 6:** Mean RFA values changes between groups and time periods up to 6 months after implant placement

	Implant placement—1 month	Implant placement—3 months	Implant placement—6 months
	N Mean (SE) [95% CI]	N Mean (SE) [95% CI]	N Mean (SE) [95% CI]
Piezo	25 −1.94 (2.77) [−7.67;3.79]	25 2.98 (2.05) [−1.24;7.20]	25 3.94 (2.11) [−0.41;8.29]
Drill	25 −0.14 (2.18) [−4.65;4.37]	25 2.58 (2.05) [−1.66;6.82]	25 3.7 (2.06) [−0.56;7.96]
Difference* [95% CI]	1.71 [−1.15;4.56]	−0.51 (−2.10;1.09)	−0.35 [−1.95;1.25]
P-value*	0.254	0.540	0.672

^*^Two-level mixed effect with patient as random effect at 1, 3 and 6 months after implant placement with baseline (implant placement) as a covariate. All changes from baseline (implant placement) non-statistically significant

## Discussion

This trial was designed to evaluate whether it could be more advantageous to prepare implant sites with piezosurgery when compared to conventional drilling. Apart from some statistically significant differences in peri-implant bone level/loss favouring drilling in the range of 0.14 to 0.17 mm observed at different timepoints, but not having a clinically significant impact, the only statistical difference having a clinical significance was the need of about 4 min more to finalise the implantation procedure using piezosurgery. Despite the fact that some may not consider this difference as clinically relevant, we cannot see any advantage in prolonging of 4 min the surgical time requested to complete the implantation procedure at each implant site using piezosurgery, especially in case of placement of multiple implants.

Our findings are, generally speaking, in agreement with those of other RCTs testing similar hypotheses. No difference for implant success rates and peri-implant bone loss was observed in a large multicentre RCT of split-mouth design [6]. No difference in implants stability measured with RFA was found in another split-mouth RCT [7]. Also another more detailed split-mouth RCT found no significant differences in peri-implant bone loss with the exception of a bit more pain perceived after 2 and 7 days at drilled sites [8]. A study, possibly randomized and of parallel group design, reported longer preparation time with piezosurgery but less postoperative pain at day 1 and 2 as well as less swelling at day one and no differences thereafter, and no differences in bone loss [9]. A split-mouth RCT comparing conventional drilling versus conventional drilling plus piezosurgery (the last 2 tips), found the only statistically significant difference at 8 weeks post-implantation for stability assessed with RFA favouring conventional drilling plus piezosurgery and no difference in radiographic bone loss 1 year after loading [2]. However, such a difference in stability may not have a clinical impact. Another similar trial found no difference as well [10] but significantly longer preparation times using piezosurgery.

One limitation of this study is the strict inclusion criteria, which focused on a homogeneous sample of healthy patients with no significant systemic diseases or compromised bone conditions. While this approach minimized confounding factors and provided a controlled environment for comparison, it also limits the broader applicability of our findings. Given that no measurable advantage was found, we believe that there is currently no compelling rationale to extend this study to populations with compromised bone quality or systemic conditions (e.g., irradiated patients, patients with diabetes, or cardiovascular disease). We do acknowledge, however, that future studies could explore piezosurgery in different patient groups, particularly those with more complex clinical conditions, to determine whether there are specific benefits for these populations.

Our findings are in disagreement with the findings from the group who invented piezosurgery [1], who presented a significantly higher loss in percentage of implant stability measured with RFA in favour of piezosurgery up to 56 days post-implantation, without evaluating other outcome measures, as well as with the findings of another group [11]. Another, possibly split-mouth, RCT reported higher implant stability values assessed with RFA favouring piezosurgery prepared sites, no differences in peri-implant bone loss and bone density, and an average of 2 min more needed when using piezosurgery [12].

Another split-mouth RCT compared piezosurgery versus rotary drills for placing zygomatic implants [13]. Zygomatic implants, as the name suggests, are placed in the zygomatic bone, that is harder than maxillary bone. While the post-surgical haematoma was larger at drilled sites, the preparation of 10% of the sites allocated to piezosurgery was unsuccessful, so drills had to be used instead. This suggests that a combined use of conventional drills and piezosurgery, when indicated, could be advantageous at least for the placement of zygomatic implants.

No particular advantages were observed when using piezosurgery as an alternative to rotary drills to prepare implant sites, however, in the proximity of the alveolar inferior nerve, piezosurgery might be an interesting alternative since potentially less damaging than conventional drilling, however, this hypothesis should be tested in properly designed and conducted RCTs.

Regarding the secondary hypothesis tested in this study, i.e. to evaluate whether crestal mucosa thickness could have an effect on bone loss, we found no effect. Our findings are in agreement with the findings/conclusions of some studies [14–16], but in disagreement with other similar studies [17–22]. These differences may be partially explained by the relatively modest variation in mucosal thickness in our study, which ranged from 1.5 to 5 mm, with only five sites having a thickness of 1.5 mm; by the long-term data from our study, compared to the short-term data typically published; and by the methodological flaws and biases affecting the majority of studies on this topic.

The main limitations of the present trial were not having investigated the patients’ post-implantation view/preference about the two different preparation techniques, and the strict inclusion criteria which may limit the generalisability of the results, for instance, to smokers. Nevertheless, since both procedures were tested in real clinical conditions, results can be generalized with confidence to a wider population having similar characteristics.

## Conclusions

No clinically appreciable differences were noticed when placing implants with piezosurgery or rotary instruments; however, piezosurgery required on average 4 min more than conventional drills. No effect of initial crestal soft tissue thickness was observed on peri-implant bone loss.

## Data Availability

No datasets were generated or analysed during the current study.
